# Impact of nicotine replacement therapy as an adjunct to anti-tuberculosis treatment and behaviour change counselling in newly diagnosed pulmonary tuberculosis patients: an open-label, randomised controlled trial

**DOI:** 10.1038/s41598-018-26990-5

**Published:** 2018-06-11

**Authors:** Surendra Kumar Sharma, Alladi Mohan, Achintya Dinesh Singh, Hridesh Mishra, Sonali Jhanjee, Ravindra Mohan Pandey, Binit Kumar Singh, Rohini Sharma, Prakash Babu Pallipamu, Madhukar Pai, Keertan Dheda

**Affiliations:** 1 Department of Molecular Medicine, Jamia Hamdard institute of Molecular Medicine, New Delhi, 110062 India; 2 Department of Medicine, All India Institute of Medical Sciences, New Delhi, 110029 India; 3 Department of Medicine, Sri Venkateshwara Institute of Medical Sciences, Tirupati, 517507 Andhra Pradesh India; 4 Psychiatry, All India Institute of Medical Sciences, New Delhi, 110029 India; 5 Biostatistics, All India Institute of Medical Sciences, New Delhi, 110029 India; 6 Canada Research Chair in Epidemiology & Global Health, Director, McGill Global Health Programs, Associate Director, McGill International TB Centre, McGill University, Dept of Epidemiology & Biostatistics, 1020 Pine Ave, West Montreal, QC H3A 1A2 Canada; 7 Lung Infection and Immunity Unit, Department of Medicine, Division of Pulmonology and UCT Lung Institute, University of Cape Town, Old Main Building, Groote Schuur Hospital, Observatory, Cape Town, South Africa; 8 Department of General Medicine & Pulmonary Medicine, JNMC, Datta Meghe Institute of Medical Sciences (DMIMS), Sawangi (Meghe), Wardha, 442004 Maharashtra India

## Abstract

We evaluated the impact of intensive smoking cessation activities as an adjunct to anti-tuberculosis treatment on patient-related treatment outcomes. In this open-label, randomised controlled trial, self-reporting smokers with pulmonary tuberculosis who initiated standard anti-tuberculosis treatment were randomised to either nicotine replacement therapy and behaviour change counselling (n = 400) or counselling alone (n = 400) provided at baseline and two follow-up visits. The primary outcomes were change in TBscore at 24-weeks and culture conversion at 8-weeks. Biochemical smoking quit rates defined as serum cotinine levels <10 ng/mL and/or exhaled carbon monoxide levels <6 ppm (47·8% vs 32·4%, p-value =< 0·001) and self-reported quit rates (69.3% vs 38·7%, p-value =< 0·001) were significantly higher in the intervention arm at 24-weeks. Though the TBscores at 24 weeks (95% CI) were lower in the intervention arm [2·07 (1·98, 2·17) versus 2.12 (2·02, 2·21)], the difference was not clinically meaningful. Patients in the control arm required treatment extension more often than intervention arm (6·4% vs 2·6%, p-value = 0·02). Combining nicotine replacement therapy with behaviour change counselling resulted in significantly higher quit rates and lower cotinine levels, however, impact on patient-related (TBscore) or microbiological outcomes (culture conversion) were not seen.

## Introduction

Tuberculosis (TB) is a major cause of global morbidity and mortality with an estimated 1.3 million TB deaths among human immunodeficiency virus (HIV) negative people and 10·4 million incident cases in 2016^1^. Among 22 high TB burden countries, India contributes to over one-quarter of the global TB burden^1^. Tobacco smoking the second largest contributor to global disability-adjusted life years after systolic hypertension^2^, is highly prevalent in the middle and low-income countries, and about 12·4% of adult population (21·9% in adult males and 2·3% in adult females) in India are estimated to be smokers^3^ with prevalence as high as 70% in some states^4^.

Smoking is strongly associated with TB disease and has a substantial population-attributable risk for TB infection, active TB disease, and mortality. Adult smokers and individuals exposed to second-hand smoke have a higher risk of acquiring pulmonary tuberculosis (PTB) than non-smokers, and the odds are even greater in heavy smokers^5,6^. Also smokers have delayed sputum culture conversion, frequently require treatment extensions, have higher treatment failure and relapse rates and greater absolute mortality as compared to non-smokers^7–9^.

Intuitively, integrating smoking cessation strategies with anti-tuberculosis treatment (ATT) should have a beneficial impact on TB treatment-related outcomes. Intensive smoking cessation methods like nicotine replacement therapy (NRT) by increasing smoking abstinence during ATT, should yield better treatment outcomes. A recent Cochrane systematic review has expressed concern over the lack of high quality evidence to endorse integration of smoking cessation activities along with ATT^10^. This is a critical unmet need because investment of substantial resources into smoking cessation interventions can only be justified and implemented if there are proven benefits to patient important outcomes.

To address this knowledge gap, the present study was carried out with the objective of studying the impact of NRT along with behaviour change counselling for smoking cessation on TB treatment-related outcomes in a controlled, clinical trial. The control arm received only behaviour change counselling along with anti-TB therapy.

## Results

### Study subjects

**Figure 1:**
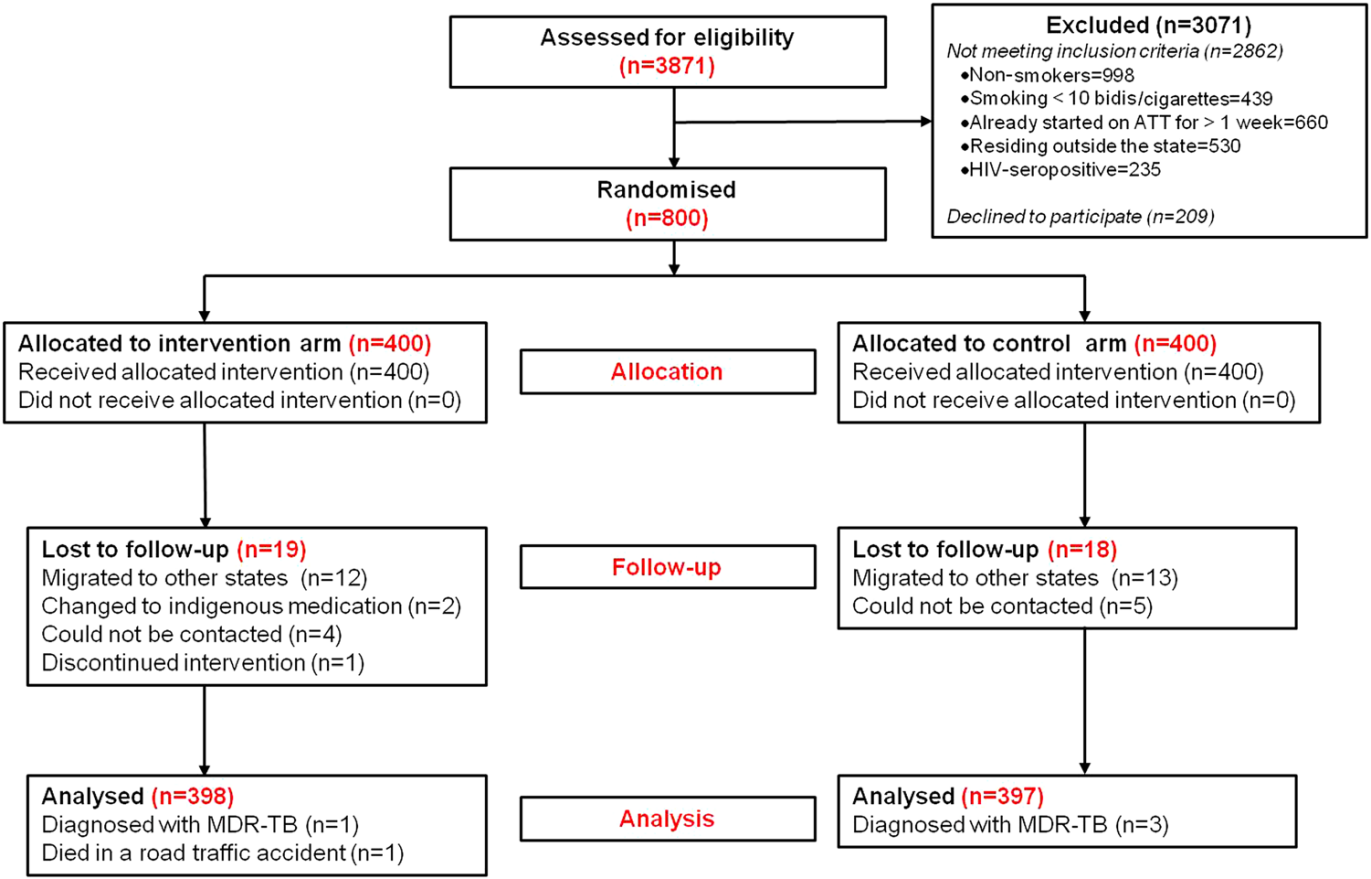
Trial profile. ATT = anti-tuberculosis treatment; HIV = human immunodeficiency virus; MDR-TB = multidrug-resistant tuberculosis.

The trial was conducted from 13 November 2010 to 03 September 2016. Of the 800 patients enrolled, one patient in the intervention arm and three patients in the control arm was later diagnosed as multidrug-resistant TB and one enrolled patient (intervention arm) died in a road traffic accident, 37 patients were lost to follow-up (Fig. 1).

### Baseline Characteristics

Table 1 details baseline characteristics of the participants. All characteristics were comparable at baseline. Majority of the patients were male. Four patients (two in each arm) were sputum smear positive but culture negative at baseline. These patients were not excluded from the study as their culture reports only became available after four weeks of enrolment.

**Table 1:** Comparison of baseline characteristics between the two arms.

Characteristics	NRT + counselling (n = 400)	Counselling alone (n = 400)
Age (years)	34·9 ± 11·9	34·23 ± 11·9
Gender		
Male	398	400
Female	02	00
Smoking pack-years	5·13 ± 7·1	4·89 ± 6·9
Breath analysis test(ppm)	17·79 ± 6·6	17·48 ± 7·1
FTND score	6·85 ± 1·1	6·73 ± 1·1
Serum cotinine level (ng/mL)	97·22 ± 5·3	97·55 ± 5·6
Alcohol intake		
Non-alcoholic	281(70·2%)	285(71·2%)
Alcoholic	119(29·8%)	115(28·8%)
Body mass index (kg/m^2^)	18·72 ± 2·6	18·39 ± 2·6
Radiological findings		
Normal	151(37·8%)	137(34·3%)
Unilateral	244(61·0%)	256(64·0%)
Bilateral	5(1·2%)	7(1·8%)
Radiological severity		
Minimal lesion	15(3·7%)	24(6·0%)
Moderately-advanced	264(66·0%)	257(64·2%)
Far-advanced	121(30·2%)	119(29·7%)
Cavitary lesion		
Cavitary	8(2·0%)	9(2·2%)
Non-cavitary	392(98·0%)	391(97·7%)
Baseline		
Negative	00	00
Scanty	163(40·7%)	162(40·5%)
1+	130(32·5%)	148(37·0)
2+	92(23·0%)	71(17·7%)
3+	15(3·7%)	19(4·7%)
TBscore at baseline	7·87 ± 1·2	7·84 ± 1·3

Values are presented as mean ± SD, categorical variables are presented in frequencies (percentage)· Abbreviations: NRT = nicotine replacement therapy, BMI = Body mass index, FTND = Fagerstrom test for nicotine dependence, Definition: Bidi = Hand rolled cigarettes containing tobacco wrapped in a tendu or temburni leaf, Smoking pack year = (Number of bidi/cigarette smoked per day × Number of years smoked)/20 (1 pack has 20 cigarettes/bidis) TBscore = validated, composite marker of tuberculosis related clinical outcome· Difference in various parameters between the two arms were not statistically significant.

### Endpoint and follow-up

#### Primary outcome

**Figure 2:**
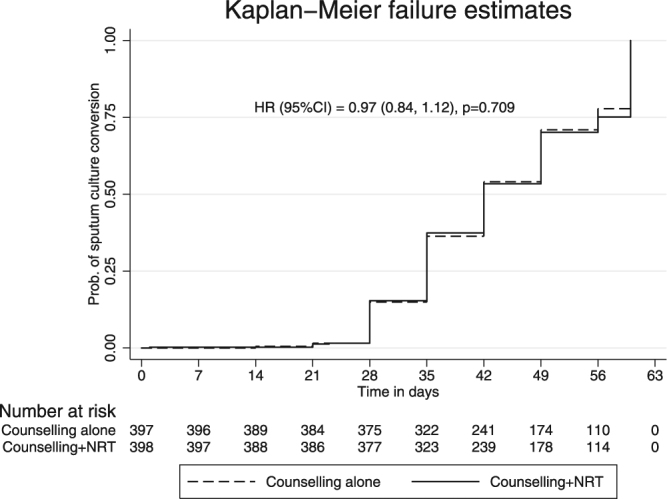
Kaplan-Meier curve showing sputum culture conversion Footnotes: HR- Hazard ratio, C·I = confidence interval, NRT = nicotine replacement therapy.

Modified intention to treat (mITT) analysis showed significant decline in TBscore from baseline to eighth week in both the arms. The decline in the intervention arm was significantly higher (difference in decline = 0·29, 95%CI = 0·007, 0·59; p value = 0·04). The direction of change and magnitude of difference were similar in per protocol analysis (0·32 95%CI = 0·058, 0·59; p value = 0·016). However, the minimal clinically important difference (MCID) was not achieved. The TBscore at 24-week was similar in both the arms (intervention arm = 2.38 ± 1·6, control arm = 2.37 ± 1·5, difference = 0·006, 95% CI = −0·22, 0·21; p value = 0·95). Sputum culture conversion was achieved in all the patients in both the arms at 8 weeks (Table 2). There was no statistical difference in sputum culture conversion rates (Fig. 2) in both the arms (Hazard ratio: 0·97, 95%C.I = 0·84, 1·12; p value = 0.709). Details of weekly sputum culture conversion: Supplement Table s3.

**Table 2:** Comparison of various outcomes between the two arms.

Outcomes	Counselling+ NRT	Counselling alone	Difference (95% C.I.)	p value
**mITT analysis**				
**Primary outcome** **:**				
**TB score**				
n Baseline Eight week Change 24 weeks Change	398 7·87 ± 1·2 3·10 ± 1·9 4·76 ± 2·05 2·38 ± 1·6 5·48 ± 1·8	397 7·82 ± 1·3 3·36 ± 1·9 4·46 ± 2·12 2·37 ± 1·5 5·45 ± 1·8	−0·04 (−0·22, 0·13) 0·25 (−0·02, 0·52) 0·29 (0·007, 0·59) 0·006 (0·22, −0·21) 0·039 (0·29, −0·21)	0·61 0·07 0·045 0·95 0.72
**Sputum culture conversion**				
n Baseline 8-weeks	398 02(0·5%) 379(95·2%)	397 02(0·5%) 379(95·5%)	— —	1·00 1·00
**Per Protocol analysis**				
**TBscore**				
n Baseline 8-weeks Change 24-weeks Change	379 7·84 ± 1·19 2·84 ± 1·60 5·00 ± 1·79 2·07 ± 0·94 5·76 ± 1·34	379 7·83 ± 1·32 3·15 ± 1·72 4·67 ± 1·92 2·12 ± 0·96 5·70 ± 1·43	0·01(0·16, −0·19) 0·31(0·07, 0·54) 0·32(0·058, 0·59) 0·04(−0·09, 0·17) 0·05(−0·14, 0·25)	0·88 0·010 0·016 0·54 0·58
**Sputum culture conversion**				
n Baseline 8-weeks	379 02 379 (100%)	379 02 379(100%)	— —	1·000 1·000
**Secondary Outcome**				
**Sputum smear Conversion**				
n Baseline 8-weeks	378 00 378	378 00 379	— —	1·000
**Weight (kg)(Mean ± SD)**				
Baseline 24-weeks Change	49·45 ± 6·9 53·47 ± 6·9 3·1 ± 2·3	50·22 ± 7·3 52·58 ± 6·5 3·1 ± 1·9	−0·78(−1·8, 0·2) 0·04(−0·2, 0·3) −0·88(−1·8,0·07)	0·120 0·070 0·760
Self-reported quit rate*				
n Eight week 24-weeks	379 222(58·5%) 263(69·3%)	379 91(24·01%) 147(38·7%)	0·4(0·29, 0·42) 0·3(0·23, 0·36)	<0·001 <0·001
**Biochemical quit rate**				
Eight week 24-weeks	73(19·3%) 180(47·8%)	47(12·5%) 121(32·4%)	0·07(0·02, 0·12) 0·15(0·08, 0·21)	<0·001 <0·001

One patient from the counselling + NRT arm and three from the counselling arm were excluded from the analysis as they were diagnosed with multidrug-resistant tuberculosis infection, one patient in the counselling + NRT arm died in a road traffic accident before the first follow-up and was excluded from the analysis. Abbreviations: mITT = modified intention to treat, ATT = Anti-tuberculosis therapy NRT = nicotine replacement therapy, C.I. = confidencce interval. Data are presented as mean ± SD· Values of significance assessed using Student’s t-test, TBscore = validated, composite marker of tuberculosis related clinical outcome, *Self reported, Biochemical quit rates = patients with serum cotinine <10 ng/mL and/or Breath analysis test <6 parts per million.

#### Secondary outcomes

**Figure 3:**
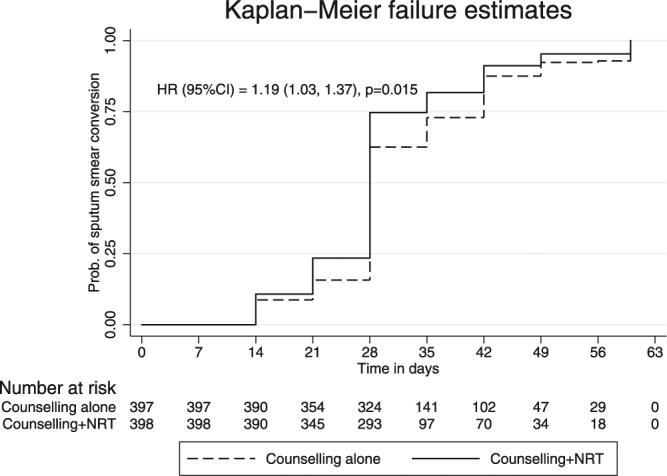
Kaplan-Meier curve showing sputum smear conversion Footnotes: HR = Hazard ratio, C·I = confidence interval, NRT = nicotine replacement therapy.

All patients in both the groups achieved negative sputum smear results by the end of 8 weeks. On weekly follow-up, 237 patients (61.7%) in the intervention arm were sputum smear negative and 51(13.2%) patients were not producing sputum at fourth week, while 206(53·7%) patients were sputum smear negative and 35(9·1%) were not producing sputum in the control arm, (p value = 0·001). In the fifth-week significantly higher proportion of patients in the intervention arm had negative sputum smear or were not producing sputum than control arm (p value = 0.01). All patients were sputum smear negative at the end of seventh week. Time to sputum smear conversion was early in the intervention arm (hazard ratio = 1·13, 95%C.I = 1·03, 1·37; p value = 0·015 Fig. 3). As per the decision of the treating medical officers, 10 patients (2·6%) in the intervention arm required treatment extension at the end of six months for three more months compared to 24 patients (6·4%) in the control arm (p value = 0·02). Eleven patients (2·9%) in the intervention arm and 12 patients (3·2%) in the control arm had relapse and were started Category II ATT as per Revised National Tuberculosis Control Programme (RNTCP) guidelines. Median time to relapse was 3 years (IQR = 2–3) in the intervention arm and 1·5 years in control arm (IQR = 0·8–2) (0·95, 95% CI = 0·24, 1·74; p value = 0·011).

The self-reported quit rates (69·3% vs 38·7%, p value < 0·001) as well as biochemical quitting (47.8% vs 32.4%, p value < 0.001) at the end of 24-week (Table 2) were higher in intervention arm than control arm. The mean reduction in serum cotinine levels, BAT results and FTND scores were also significantly greater in the intervention arm (Table 3). Increase in body weight across both the arms at the end of 24-week was similar (−0·88, C.I = −1·8, 0·07, p value = 0·76) and subsequent to treatment completion most patients in both arms reported re-initiation of tobacco smoking (80·6% vs 79·7%).

**Table 3:** Comparison of serum cotinine levels, breath analysis test, FTND levels between the two arms.

Test	Counselling+ NRT		Counselling alone		Difference (95% C·I.)	p value
	n	Mean ± SD	N	Mean ± SD		
**Serum cotinine levels (ng/mL)**						
Baseline	328	97·24 ± 5·3	320	97·52 ± 5·59	0·27 (−0·56, 1·12)	0·510
2nd week	317	86·06 ± 15·5	311	90·60 ± 13·87	4·53 (2·23, 6·84)	0·001
4th week	314	71·87 ± 22·7	305	78·38 ± 18·01	6·54 (3·26, 9·74)	0·001
2nd month	309	57·86 ± 28·1	300	66·63 ± 21·9	8·77 (4·75, 12·79)	0·001
6^th^ month	307	45·87 ± 32·7	297	55·12 ± 27·31	9·25 (4·42, 14·07)	0·002
Total change		51·48 ± 33·5		43·36 ± 27·09	9·11 (4·23, 13·99)	0·0003
**BAT (ppm)**						
Baseline	387	17·82 ± 6·68	391	17·52 ± 7·13	−0·30 (−1·27, 0·66)	0·540
2nd week	375	14·19 ± 6·12	380	14·24 ± 5·48	0·005 (−0·78, 0·88)	0·900
4th week	371	11·21 ± 4·85	374	11·25 ± 4·37	0·04 (−0·62, 0·71)	0·890
2nd month	368	9·39 ± 3·95	370	10·12 ± 5·62	0·73 (0·029, 1·43)	0·040
6th month	375	7·82 ± 3·29	372	8·51 ± 3·2	0·69 (0·22, 1·15)	0·003
Total change		9·8 ± 6·2		8·8 ± 6·9	0·94 (0·14, 1·90)	0·053
**FTND**						
Baseline	398	6·85 ± 1·06	397	6·73 ± 1·13	−0·11 (0·27, 0·34)	0·120
2nd week	387	4·49 ± 1·39	390	4·93 ± 1·36	0·44 (0·24, 0·63)	0·001
4th week	384	2·84 ± 1·58	384	3·46 ± 1·5	0·62 (0·40, 0·84)	0·001
2nd month	380	1·58 ± 1·4	380	2·54 ± 1·52	0·96 (0·75, 1·16)	0·001
6th month	375	1·38 ± 1·25	375	2·13 ± 1·34	0·75 (0·56,·93)	0·001
Total change		5·45 ± 1·6		4·61 ± 1·8	0·83 (0·59, 1·07)	0·001

mITT = modified intention to treat, ATT = Anti-tuberculosis therapy, NRT = nicotine replacement therapy BAT = Breath analysis test, FTND = Fagerstrom Test for Nicotine Dependence, C.I. = confidencce interval.

## Discussion

This is the first randomised trial to holistically assess the impact of combined NRT with behaviour change counselling on smoking cessation and its role in improving TB treatment outcomes. The key finding of this study is that although intervention of NRT combined with counselling achieved higher smoking cessation and quit rates and a greater reduction in TB score was seen in intervention arm, the difference was below the MCID, and both the arms had similar sputum culture conversion rates. However, the intervention arm had earlier sputum smear conversion beginning from the third week up to the fifth week. Also, treatment extension rates were higher in the control arm compared to the intervention arm.

TBscore, the primary outcome, did not meaningfully change at 8 and 24 weeks. NRT was provided only for the initial six weeks after which both the arms received the same counselling. The reduction in smoking seen across both the arms may have contributed to the similar decline of TB score. The mean scores at the end of 8-weeks in our study (intervention arm = 3·1 ± 1.9, control arm = 3·3 ± 1.9) are similar to the original study on TBscore by Wejse *et al*. (3·0 ± 2.3) providing credibility to our results^11^. Although we could not show impact using proxy measures (TBscore) we wondered if a larger study could have shown an impact on outcomes such as treatment failure, cure rates, and relapse? This seems less likely as our statistical calculations were robust and conservative (powered to detect a 0·5 point difference in the TBscore) and we were able to show significant improvement in quit rates between the arms.

Though various studies have shown an association between smoking and delayed culture conversion^7,8^, the impact of smoking cessation on TB treatment outcomes has never been conclusively demonstrated. Shaler *et al*. found continuous and not discontinuous exposure to cigarette smoke the major cause of impediment of immune function^12^. The mean decline of smoking across both the arms may have had led to immune recovery in both the arms. It is possible that we could have observed a significant difference in the TBscore and sputum culture conversion during the intensive phase of treatment if, one of the arms had ‘usual standard of care’ which is what occurs in routine clinical practice where no counselling is provided. However, having such an arm was deemed unethical by our research committees. We therefore propose that behaviour change counselling should be an integral part of the National Tuberculosis Program similar to pre-test and post-test counselling done in HIV/ AIDS. Healthcare providers should be adequately trained to acquire counselling skills.

A recent study by Awaisu *et al*. found a trend towards earlier sputum smear conversions at 8-weeks with higher sputum smear negativity at 24 weeks in the intervention group receiving counselling and NRT (100% vs 93·9%)^13^. However, this study^13^ had several limitations including a non-randomised study design resulting in selection bias, high drop-out rates during the course of the study, non-uniformity of the baseline characteristics between the groups, and Hawthorne effect in the intervention arm. Also, only smokers who were motivated to quit smoking were enrolled in the intervention group and drug therapy was modified as per individual patient’s adherence to smoking cessation. Due to these drawbacks, findings of that study cannot be generalized to smokers. Nonetheless, the trend of early sputum smear conversion shown in that study provides corroborative evidence to the impact of interventions on sputum smear results observed in our study.

In the present trial, delayed radiological improvement and treatment extensions were more frequent in control arm than intervention arm (6·4% vs 2·6%, p value = 0·02). The median time to relapse in the intervention arm was 3 years as compared to 1·5 years in control arm. These results are congruent with previously reported studies implicating the role of continued smoking in delayed improvement, treatment extension and relapse^7,8,13,14^.

The biochemical quit rates found in our study at six months were 47.8% and 32·4% in the intervention and control arms, respectively. Both, serum cotinine and exhaled breath carbon monoxide levels were measured to avoid false negative results due to omissions and negative self-reporting^15^. Assessment of BAT levels accounted for any false elevation in serum cotinine levels due to smokeless tobacco use or nicotine replacement therapy^16^. These results are similar to a recent study^17^ in smokers with TB which found abstinence rates of 71·7% in the group receiving counselling along with slow-release bupropion as compared to counselling alone (33·9%), and a no counselling group (9·8%).The authors did not report the impact on treatment-related outcomes. Our findings also receive credence from a recent Cochrane systematic review, which concluded that combined nicotine replacement and counselling methods are better for smoking cessation than a casual or brief advice or no intervention^18^. Patients concerns about their illness and desire to achieve faster recovery can itself motivate them to quit smoking^19^. This, along with focused counselling led to reduction in all smoking-related parameters across both the arms. However, a greater reduction in the intervention arm may be attributed to the role of NRT administration in further boosting their motivation levels by ameliorating the cravings associated with nicotine addiction^20^. On telephonic enquiry, high rate of re-initiation of tobacco smoking in both arms after completion of therapy, emphasises the need for continuous reinforcement of counselling in the highly smoking dependent group. This was not the objective of the study as our smoking cessation interventions were provided only up to six weeks.

The trial has many strengths. Adherence to the smoking cessation strategies was monitored by subjective as well as objective assessments. Outcomes were assessed using clinical parameters as well as culture and sputum smear techniques. TBscore was found to be an effective tool for clinical assessment and can be incorporated for monitoring of patients on anti-TB therapy. The trial was conducted in several DOT centres of two trial sites under RNTCP and demonstrates the feasibility of integrating smoking cessation strategy into the national TB control programmes. The study has wide applicability globally to combat ongoing and colliding epidemics of tobacco smoking and TB. However, a number of limitations should be noted. The trial did not have a “drug treatment only” arm without counselling. Also, the results of the study are ascribable to predominantly a single centre. The absent sputum production in a significant proportion of patients led to high loss to follow-up of the culture reports. Socio-economic factors like literacy, monthly income which can confound the effects of smoking cessation methods on quitting were not noted during the study.

Overall our findings suggest that counselling alone was also found to be feasible and effective method for smoking cessation, however combination therapy had higher compliance. Though the clinical improvement seen in the study was not meaningful, the greater reductions seen in the NRT with counselling arm could have been significant if compared against a control arm likely to be seen in routine clinical practice. Larger pragmatic studies are now required to determine if smoking cessation interventions can impact important clinical outcomes such as treatment failure, cure, relapse and mortality.

## Methods

### Study design and Participants

We conducted a two-centre open-label, randomised, controlled, clinical trial in India. Trial centres were at the All India Institute of Medical Sciences (AIIMS), New Delhi and neighbouring tuberculosis and chest clinics in the National Capital Region of Delhi and the Sri Venkateswara Institute of Medical sciences (SVIMS), Tirupati, Andhra Pradesh.

All adult patients (>18years) with newly diagnosed sputum-positive pulmonary tuberculosis with self-reported history of current cigarette/rolled tendu or temburni leaf (bidi) smoking (more than 10 per day, every day for at least two months), providing written informed consent were recruited by invitation.

Monoresistant TB, multidrug-resistant TB, extensively drug-resistant TB cases^21^ and patients who had received ATT for more than 1-week were excluded from the trial. Patients with known contraindications to the use of NRT like myocardial infarction within last six months or history of peripheral vascular disease were excluded from the study. Patients with asthma, depression, on immunosuppressive therapy and HIV-TB co-infected patients were also excluded from the study. Ethical clearance was obtained from AIIMS, New Delhi institutional ethics committee and SVIMS, Tirupati institutional ethics committee. The trial was conducted as per Good Clinical Practice guidelines. The trial is registered at ClincalTrials.gov: NCT01517022 on 23/06/2011. Protocol available at: https://clinicaltrials.gov/ct2/show/NCT01517022.

### Randomisation

Patients were randomised in a 1:1 ratio to the arm receiving behaviour change counselling with NRT in the form of nicotine chewing gums (intervention arm) or only behaviour change counselling (control arm). Randomisation was performed by generation of random numbers through sequentially numbered, opaque sealed envelopes using a block randomisation scheme with variable block size.

### Procedures

All the patients, irrespective of the allocation, received intermittent, ATT as per the RNTCP guidelines which supplies drugs free-of-cost on an alternate-day schedule. During the initial Intensive Phase, isoniazid(H), rifampicin(R), ethambutol(E) and pyrazinamide(Z) were provided thrice weekly on every alternate day for two months under direct supervision of the healthcare worker for newly diagnosed pulmonary TB patients^22^. It was followed by a Continuation Phase of H and R thrice weekly for the next four months. In this phase, the first dose of the week was directly supervised and the remaining two doses were self-administered by patients.

The intervention package for smoking cessation comprised:

#### Behaviour change counselling

Patients in both arms were provided with individual behavioural counselling at baseline (day of enrolment in the study), second and fourth week, each session lasted for around 10 minutes. It was designed to enhance awareness about the harmful effects of smoking on health, economy and on other family members. They were provided with problem solving/skills training techniques to handle craving, withdrawal, and avoid relapse. Information was reinforced by providing pamphlets and educational materials at each follow-up visit. The educational materials prepared by The Union, American College of Chest Physicians and “Cease smoking today” guidelines were used after translating into local languages (Hindi, Punjabi and Telugu)^23–25^. Counselling was imparted by healthcare workers who were trained by an expert (psychiatrist) in smoking cessation over two days. Their efficiency was periodically assessed by the investigators and retraining was provided if deemed necessary.

#### Nicotine replacement therapy (NRT)

Patients in the intervention arm were also provided with nicotine chewing gums (nicogum) of two mg or four mg for six weeks. Two mg nicogum was administered to patients smoking up to 25 bidis/cigarettes/day, whereas four mg nicogum was given to patients smoking more than 25 bidis/cigarettes/day. They were provided the chewing gums at baseline, the second week visit and the fourth week visit. They were advised to chew the gum till they get a flavour, then park it between the cheek and gums, and to restart chewing once the taste fades. Patients were instructed to use the gum at fixed intervals throughout the day and if there was additional craving; use of more than 24 gums in a day was discouraged^26^.

### Outcome assessment

TBscore was assessed by the medical healthcare workers under the supervision of doctors posted in various trial centres on the 8-weeks and 24-weeks follow-up visits (Supplement Table s1). TBscore assesses five symptoms namely cough, hemoptysis, dyspnea, chest pain and night sweats. Each of these symptoms if present are given one point. Objective assessments include anemic conjunctiva, tachycardia (heart rate more than 100), any positive findings on lung auscultation, body mass index (BMI) less than 18 kg/m^2^, axillary temperature (more than 38 °C) and mid upper arm circumference (MUAC) less than 220 mm. All of these parameters were provided one point each. If the BMI was less than 16.5 kg/m^2^ or the MUAC was less than 200 mm the patients were provided an additional one point. The TBscore correlates with disease severity and also predicts mortality due to TB^11,27^.

Sputum smear microscopy for acid-fast bacilli was done at baseline and at each follow-up visit for all patients who were producing sputum. Sputum induction techniques were not used. Each slide was made directly from one sample; staining, microscopy and bacillary grading were done as per standard RNTCP guidelines^28^.

Sputum culture using solid and liquid culture media were done at baseline, 8-weeks and 24-weeks. Processed samples were inoculated into MGIT-960 non-radiometric automated isolation system. Positive cultures were confirmed using immunochromatographic assay kit^29^. Baseline *in-vitro* anti-TB drug susceptibility testing (DST) for isoniazid H, R, E and S was done for all patients by inoculation on Lowenstein-Jensen media as per the standard operating procedure^30^ or inoculation on BACTEC MGIT-960 system as per the standard operating procedure^29^. Susceptibility testing for Z was not done, as it is technically difficult and the gold standard technique for Z resistance^31^ was outside the purview of this study. GenoType MTBDRplus line probe assay was also performed as per the World Health Organization (WHO) recommendations on all culture positive isolates^32^.

### Smoking cessation parameters

Smoking cessation was assessed both subjectively and objectively at all follow-up visits. Serum cotinine levels were measured using enzyme-linked immunosorbent assay method. Breath analysis test (BAT) assessing the exhaled carbon monoxide level was done employing PICO Smoke analyser carbon monoxide monitor (Technical details supplement Table: s2). Fagerstrom test for nicotine dependence (FTND)^33^ assessed subjective dependence on smoking. A chest X-ray was performed at baseline and at 24 weeks follow-up visit and the pulmonary involvement was classified as described previously^34^.

### Follow-up

Patients were followed-up at 2-weeks, 4-weeks, 8-weeks and 24-weeks at their nearest TB centre/chest clinic. In case of loss to follow-up, patients were telephonically contacted or the healthcare workers visited their homes to persuade them back into the study. After treatment completion, patients were telephonically contacted every 4–6 months about their general health status, smoking habits, etc. Also the patients were advised to contact the healthcare workers if they experienced recurrence of symptoms.

### Outcome

The primary outcome was a change in the TBscore at 24-weeks and sputum culture conversion at eight-week. Secondary outcomes were time to sputum smear conversion, weight gain at 24 weeks, number of patients who have quit smoking by 24-week, and mortality by 24-week. WHO guidelines were used for defining treatment failure rates, cure rates and default rates^21^. Quit rates were both self-reported smoking abstinence and biochemical quit rates defined as serum cotinine levels less than 10 ng/mL or BAT less than six parts per million.

Patients enrolled in the study after January 2014 at the AIIMS centre were followed-up on a weekly basis till the eight-week with sputum culture and smear tests. This ensured detecting early sputum culture and smear conversions but did not influence the originally decided outcome in any manner. The original protocol was continued at the second centre, SVIMS.

### Statistical analysis

The sample size was calculated on the basis of the primary outcome of TBscore^11^. Wejse *et al*. found that the MCID was just above 1 point (i.e. 1 sign/symptom or a change in BMI/MUAC). Although this difference is relevant when comparing overall scores or groups of patients, the authors found that an anchor based approach of a 25% change in baseline score was more practical in terms of defining clinical improvement. We conservatively assumed that the difference between the arms will be only 0·5 point (approximately 25% reduction in the TBscore from baseline to the end of 24 weeks). We also assumed, based on previous studies, that the within group standard deviation will be two points in both arms. We needed 335 patients to achieve power of 90% with 5% two-sided level of significance. Also, to account for the early deaths, loss to follow-up and withdrawals we inflated this number by 20% and required 400 patients in each arm.

Statistical analysis was carried out using STATA 12·0 (College Station, Texas, USA). Data were presented as number (percentage) or mean ± SD/median (minimum-maximum) as appropriate. Baseline categorical variables were compared between the groups using Chi-square test/Fischer’s exact test and continuous variables were compared using Student’s ‘t’ test/Wilcoxan ranksum test. To analyse the outcomes of the study both mITT and perprotocol (pp) were carried out. The TBscore was compared using Student’s ‘t’ test. The sputum culture conversion and smear conversion were compared using z-test. The results were presented as change over time (95% CI). Hazard ratios were calculated using Cox regression analysis.

### Data availability statement

All data generated or analysed during this study are included in this published article (and its Supplementary Information files).

## Electronic supplementary material

Supplementary information
