# Supplementary material for: Impact of nicotine replacement therapy as an adjunct to anti-tuberculosis treatment and behaviour change counselling in newly diagnosed pulmonary tuberculosis patients: an open-label, randomised controlled trial
Source: Sci Rep. 2018 Jun 11;8:8828. doi: 10.1038/s41598-018-26990-5 (PMC5995820; doi:10.1038/s41598-018-26990-5)
Supplement: Supplementary file 1 — Supplementary information [file 41598_2018_26990_MOESM1_ESM.docx]

# **Impact of nicotine replacement therapy as an adjunct to anti-tuberculosis treatment and behaviour change counselling in newly diagnosed pulmonary tuberculosis patients: an open-label, randomised controlled trial**

* Surendra Kumar Sharma^1^, Alladi Mohan^2^, Achintya Dinesh Singh^3^, Hridesh Mishra^3^, Sonali Jhanjee^4^, Ravindra Mohan Pandey^5^, Binit Kumar Singh^3^, Rohini Sharma^3^, Prakash Babu Pallipamu^2^, Madhukar Pai^6^, Keertan Dheda^7^

**Institutional Affiliations**

^1^Department of Molecular Medicine, Jamia Hamdard institute of Molecular Medicine, New Delhi,110062, India

^2^Department of Medicine, Sri Venkateshwara Institute of Medical Sciences, Tirupati, Andhra Pradesh, India,

^3^Department of Medicine, All India Institute of Medical Sciences, New Delhi-110029, India,

^4^Psychiatry, All India Institute of Medical Sciences, New Delhi-110029, India,

^5^Biostatistics, All India Institute of Medical Sciences, New Delhi-110029, India,

^6^Canada Research Chair in Epidemiology & Global Health, Director, McGill Global Health Programs, Associate Director, McGill International TB Centre, McGill University, Dept of Epidemiology & Biostatistics, 1020 Pine Ave West Montreal,  QC H3A 1A2, Canada,

^7^Lung Infection and Immunity Unit, Department of Medicine, Division of Pulmonology and UCT Lung Institute, University of Cape Town, Old Main Building, Groote Schuur Hospital, Observatory, Cape Town, South Africa

* **Correspondence**

SK Sharma, MD, Ph.D., FNASc, FNA, JC Bose Fellow

Former Chief, Division of Infectious diseases

Senior Professor & Head, Department of Internal Medicine,

(WHO Collaborating Centre for Research & Training in Tuberculosis)

(Centre of Excellence for EPTB [MOH &FW, Gol])

All India Institute of Medical Sciences, New Delhi-110029, India

PH 91 11 2659 3303(O), 91 11 2659 4415(O)

FAX 91 11 26589994

Email: [sksharma.aiims@gmail.com](mailto:sksharma.aiims@gmail.com)

**Current**:

Adjunct Professor, Department of Molecular Medicine,

Jamia Hamdard institute of Molecular Medicine,

Hamdard University, Hamdard Nagar, New Delhi 110 062, India.

PH 91 11 2659 3303(O), 91 11 2659 4415(O)

FAX 91 11 26589994

E-mail: sksharma.aiims2@gmail.com;

sksharma@aiims.ac.in

| **Table s1: Showing the components comprising the Tuberculosis score** | |
| --- | --- |
| **Self-reported** | |
| 1. Cough   2. Hemoptysis  3. Dyspnoea  4. Chest Pain  5. Night Sweating | 1  1  1  1  1 |
| **Examination** | |
| 6.Anemic conjunctivae  7. Tachycardia  8.Positive findings on lung auscultation  9.Axillary temperature >38^0^c  10.BMI <18 (kg/m^2^)  11.BMI<16 (kg/m^2^)*  12.MUAC<220 mm^#^  13.MUAC<200 mm^#^ | 1    1  1  1  1  1  1  1 |
| *-Body-Mass Index less than 16 will be scored:2 #- Mid Upper arm circumference< 200mm: 2 points  Wejse C, Gustafson P, Nielsen J, Gomes VF, Aaby P, Andersen PL, et al. TBscore: Scand J Infect Dis. 2008;40(2):111–20 | |

| **Table s2: Technical details of various tests performed in the study** | |
| --- | --- |
| **Test performed** | **Technical details** |
| Nicotine Chewing gums | Nicogum, chewing gums, CIPLA LTD, Okasa Pvt. Ltd, Hingna Road, Nagpur, Maharashtra, India) |
| GenoType MTBDRplus line probe assay version-2 | Hain Lifescience, Nehren, Germany |
| Immunochromatographic assay kit | SD MPT64TB Ag kit developed by Standard Diagnostics, South Korea |
| MGIT-960 non-radiometric automated isolation system | Becton Dickinson, Sparks, MD, USA |
| Serum cotinine levels | Calbiotech ELISA kits (Spring Valley, CA, U.S.A) |
| Breath analysis test | PICO Smoke analyser carbon monoxide (CO) monitor (Bedfont Scientific Ltd, England) using disposable cardboard mouth pieces |

| **Table s3: Comparison of weekly sputum culture and sputum smear conversion between the two arms** | | | | | | | |
| --- | --- | --- | --- | --- | --- | --- | --- |
| **Time point of sputum testing** | **Results** | **Sputum culture** | | **P** | **Sputum smear** | | **p** |
|  |  | **Counselling +NRT (%)** | **Counselling alone (%)** |  | **Counselling +NRT (%)** | **Counselling alone (%)** |  |
| **Baseline** | n  Positive  Negative  NSP* | 398  396(99.5)  02(0.5)  00 | 397  395(99.5)  02(0.5)  00 | 1.000 | 398  398  00  00 | 397  397  00  00 | 1.000 |
| **1^st^ week** | n  Positive  Negative  NSP | 302  302  00  00 | 312  312  00  00 | 1.000 | 302  302  00  00 | 312  312  00  00 | 1.000 |
| **2^nd^ Week** | n  Positive  Negative  NSP | 293  292(99.6)  01(00.4)  00 | 305  302(99.0)  03(1.0)  00 | 0.620 | 389  354(91.0)  35(9)  00 | 388  345(88.9)  43(11.1)  00 | 0.340 |
| **3^rd^Week** | n  Positive  Negative  NSP | 291  286(98.3)  04(1.4)  01(0.3) | 302  294(97.4)  05(1.6)  03(0.9)) | 0.810 | 292  229(78.4)  61(20.8)  02(0.7) | 302  258(85.4)  37(12.2)  07(2.3) | 0.006 |
| **4^th^ Week** | n  Positive  Negative  NSP | 287  229(79.8)  54(18.8)  04(1.4) | 297  240(80.8)  51(17.1)  06(2.1) | 0.750 | 384  96(25.0)  237(61.7)  51(13.2) | 383  142(37.1)  206(53.7)  35(9.1) | 0.001 |
| **5^th^Week** | n  Positive  Negative  NSP | 286  143(50.0)  89(31.1)  54(18.9) | 295  157(53.2)  87(29.4)  51(17.3) | 0.730 | 287  52(18.1)  27(9.4)  208(72.5) | 297  73(24.6)  41(13.8)  183(61.6) | 0.019 |
| **6^th^Week** | n  Positive  Negative  NSP | 286  81(28.3)  69(24.12)  136(47.5) | 295  88(29.8)  72(24.4)  135(45.7) | 0.890 | 287  16(5.6)  41(14.3)  230(80.1) | 297  20(6.7)  56(18.8)  221(74.4) | 0.240 |
| **7^th^Week** | n  Positive  Negative  NSP | 286  15(5.2)  64(22.3)  207(72.3) | 294  23(7.8)  61(20.7)  210(71.4) | 0.440 | 287  00(0.0)  17(5.9)  270(94.1) | 297  00  21(7.1)  276(92.9) | 0.610 |
| **2^nd^ Month** | n  Positive  Negative  NSP | 379  00  26(6.8)  353(93.2) | 378  00  26(7.3)  352(92.7) | 1.000 | 379  00  96(25.3)  283(74.6) | 378  00  83(21.9)  295(78.1) | 0.300 |
| **6^th^ Month** | n  Positive  Negative  NSP | 379  00  00  379 | 378  00  00  378 | 1.000 | 376  00  129(34.3)  247(65.7) | 376  00  118(31.3)  258(68.7) | 0.430 |

Results are presented as frequencies (percentage). The value of significance was calculated by applying chi-square/fisher exact test among control and cessation group. Incomplete data of MDR-TB patients and lost to follow-up are excluded from the analysis. The values are presented as per the protocol of the research study encompassing follow up at second week, fourth week, eight weeks and 24 weeks after enrolment. *NSP-No sputum production, NRT-Nicotine replacement therapy.
